# Exploring diet, exercise, chronic illnesses, occupational stressors and mental well-being of healthcare professionals in Punjab, Pakistan

**DOI:** 10.1186/s13104-017-3096-5

**Published:** 2017-12-19

**Authors:** Waqas Ahmad, Ahmed Waqas, Haider Ali Saleem, Sadiq Naveed

**Affiliations:** 1 CMH Lahore Medical College & Institute of Dentistry, Lahore Cantt, Pakistan; 2 Aga Khan University, Karachi, Pakistan; 3 KVC Prairie Ridge Psychiatric Hospital, Kansas City, KS USA

**Keywords:** Wellbeing, Mental health, Healthcare professionals, Warwick Edinburgh mental well-being scale, Diet, Exercise, Stressors

## Abstract

**Objective:**

This data set was compiled to assess mental well-being, dietary pattern and physical health parameters of health care professionals in Pakistan.

**Data description:**

The Warwick-Edinburgh mental well-being scale was first evaluated for the Pakistani population then used, along with other measures like body mass index, exercise and dietary habits to assess health and wellbeing of health care providers. The importance of the data lies in the fact that no previous records or data exists in our knowledge that used a subjective index to assess wellbeing in Pakistani population. Furthermore, this data may be used as part of a global analysis to find differences in well-being and health habits of health care providers in developing countries as opposed to developed countries.

**Electronic supplementary material:**

The online version of this article (10.1186/s13104-017-3096-5) contains supplementary material, which is available to authorized users.

## Objectives

Pakistani health care professionals (HCPs) have a high prevalence of stress owing to poor work-life balance, financial insecurity, excessive dealing with death and dying and often hostility [1]. Previous studies have shown that these work associated stressors predispose them to different psychopathologies such as anxiety, depression, poor sleep quality and suicidal ideation [2, 3]. Additionally, healthcare professionals especially doctors also report poor life style, dietary and exercise habits [4].

These poor habits eventually lead to inadequate health practices by patients because a physician’s health counseling often depends on his own health habits [5]. Furthermore, patients are likely to practice habits they see in their physicians [5, 6]. Recognizing this, we conducted this cross-sectional survey to assess mental well-being and physical health of HCPs in Pakistan. This survey also reports the first use of Warwick-Edinburgh mental well-being scale (WEMWBS) in Pakistan. WEMWBS has shown good reliability and cross-cultural validity among different populations [7]. Different physical health parameters were also explored including diet, exercise, sleep, body mass index, chronic illnesses and occupational stressors. Using this data, our previous published studies explored predictors of mental well-being and validity and reliability statistics of WEMWBS among the Pakistani population [8, 9]. This dataset can be good resource for future studies exploring mediation and moderation pathways related to physical and mental well-being of healthcare professional.

## Data description

The data collected was through convenience, non-probability sampling technique. A total of 1510 questionnaires were administered to health care professionals from seven cities in the province of Punjab, Pakistan. These healthcare professionals were approached individually at their workplaces (clinics and hospitals) by a team of 16 medical students. A total of 1319 responses were received yielding a response rate of 87.35%. The study group of HCPs comprised of doctors, dentists, nurses, physiotherapists and pharmacists. The dataset provides demographic details of respondents, scores on the (WEMWBS), dietary pattern using a food frequency questionnaire, exercise pattern, occupational stressors and chronic illnesses among HCPs (Data file 1, Table 1) (Additional file 1).

**Table 1:** Dataset exploring mental well-being of healthcare professionals in Pakistan

Label	Name of data file/data set	File types (file extension)	Data repository and identifier (DOI or Accession Number)	License
Data file 1	Mental well-being of HCPs in Pakistan	SPSS	10.6084/m9.figshare.5589766	CC-BY 4.0

Demographic details include gender, city, profession and age of the respondents. This is followed by experience of occupational stressors and chronic illnesses and several physical health related characteristics of healthcare professionals. Body mass index was calculated using values of weight and height provided by the participant.

The food frequency questionnaire included intake of carbohydrates, proteins, dairy, fruits, and vegetables, divided in three categories i.e. “required”, “more than required”, “less than required”. The reference values and cutoffs used were according to USDA dietary guidelines (2010). Using the same guidelines, intake of saturated and unsaturated fats and sugars was noted in the categories of “infrequently consumed”, “often consumed” or “frequently consumed”. Water intake was noted as frequency of glasses (250 ml) consumed per day into three categories, i.e. “1–6”, “6–8”, “greater than 8”. Breakfast frequency was noted per week under “daily”, “less than 7” or “none”. Frequency of fast food consumption per week was noted in four categories i.e. “none”, “less than 5”, “five to seven” and “greater than seven”. Consumption of tea and coffee was noted in free form, with responses varying from ‘0’ to ‘10’ cups a day. Lastly, intake of nutritional supplements such as vitamin D was also noted as a dichotomous yes/no response (Additional file 2).

Exercise behavior of participants were classified into three categories, those getting the “required” amount of exercise, those with “less than required” and those with “none” at all. The required amount was defined as per guidelines by the American Heart Association i.e. 150 min per week of moderate activity or 75 min per week of vigorous activity or a combination of both.

Chronic diseases were noted as “yes” or “no”. These included coronary heart disease, hypertension, diabetes mellitus, visual defects, back problems. To evaluate perception of occupational stressors, subjective data was collected on perception of occupational stressors in workplaces. These factors included long working hours, patient overload, uncertain future, insufficient opportunities to prosper and experience of illegitimate political or administrative pressures in work place.

Personal lifestyle choices that contribute to health and well-being were inquired for including habit of smoking, routine checkups with doctors, work hours per week, amount of time spent with family (in hours per week) and ease of access to basic life needs like education and medicines. Participants were also classified in income groups of “low income”, “lower middle”, “upper middle”, “high” and “honorary” according to World Bank classification. Finally, participants were also inquired about their opinions on adequate work and career aspirations. Detailed description of methodology and questionnaires used to interview participants have been provided as additional files (Additional files 1 and 2).

## Limitations

- Convenience sampling is used therefore the extent to which the respondents are representative of the target population is uncertain.
- WEMWBS was not translated into Urdu language; the official language of Pakistan.
- Data was only collected from Punjab so its findings cannot be generalized to the whole Pakistani population.
- In absence of regional dietary, physical exercise guidelines, dietary criteria by the USDA and recommended exercise pattern by the AHA were employed.
- BMI calculations were based on values provided by participants themselves which can introduce reporting bias.
- Occupational stress was measures very subjectively by presence or absence, rather than with a cross-culturally validated scale.
- HCPs were not inquired about their psychiatric health using scales for common mental illnesses.
- Joint family systems are more common in Pakistan, however, we did not ask about the total number of household dependents which can play a role in financial wellbeing.

## Additional files

**Additional file 1.** Detailed description of methodology. This file describes detailed methodology employed in the collection of data for “Exploring diet, exercise, chronic illnesses, occupational stressors and mental well-being of healthcare professionals in Punjab, Pakistan”.

**Additional file 2.** Questionnaire used for interviewing participants. This document contains the questionnaire employed in present study.

## Abbreviations

WEMWBS: Warwick-Edinburgh mental well-being scale
HCPs: health care providers
USDA: United States Department Of Agriculture
AHA: American Heart Association
