# Supplementary material for: Exploring diet, exercise, chronic illnesses, occupational stressors and mental well-being of healthcare professionals in Punjab, Pakistan
Source: BMC Res Notes. 2017 Dec 19;10:745. doi: 10.1186/s13104-017-3096-5 (PMC5735512; doi:10.1186/s13104-017-3096-5)
Supplement: Supplementary file 1 — Additional file 1. Detailed description of methodology. This file describes detailed methodology employed in the collection of data for “Exploring diet, exercise, chronic illnesses, occupational stressors and mental well-being of healthcare professionals in Punjab, Pakistan”. [file 13104_2017_3096_MOESM1_ESM.docx]

Methodology

Following sample size recommendations made by Van Voorhis & Morgan , a total of 1,510 health care providers (HCPs) were approached conveniently to take part in the study [1]. A total of 1,319 responses were received yielding a response rate of 87.35%.

Sixteen medical students approached HCPs in both public and private clinical settings and after obtaining written informed consent administered the research questionnaires. The study sample comprised of doctors, nurses, physiotherapists, pharmacists and dentists from seven cities of Punjab, Pakistan, namely; Lahore, Faisalabad, Gujarat, Multan, Rawalpindi, Islamabad and Sheikhupura. The data were collected from March through July, 2013.

Firstly, a pilot study of fifty HCPs was completed in Combined Military Hospitals in Lahore. The purpose of this was to evaluate the comprehensibility of the research questionnaire among the HCPs. The questionnaire including the English version of the WEMWBS was found to be easily comprehensible, thus, establishing a good face validity.

The questionnaire comprised of four parts including demographic characteristics, WEWMBS, food frequency questionnaire (Table 1), exercise questionnaire, physical health parameters and work related stressors.

The socioeconomic status of participants was evaluated using the World Bank Criteria into following groups; low income (⩽8500 PKR), lower middle (between 8,501 and 33,000 PKR), upper middle income (between 33,001 and 102,000 PKR) and high (greater than 102,000).

Warwick Edinburg Mental Wellbeing scale (WEMWBS) to assess mental wellbeing as it has previously been found to be widely reliable, valid, as well received in the English speaking Pakistani Population [2,3]. In the Pakistani population, the scale has shown good psychometric properties, face validity, a high internal consistency (0.89), a unidirectional construct and a good test-retest reliability in a previous publication using the same dataset. We showed that both principle axis factoring and principal component analysis demonstrate similar factor structure of the WEMWBS [4]. The scale comprises of fourteen items that cover both the eudemonic (happiness and pleasure) and hedonic (self realization) aspects of mental wellbeing. It is scored on a one to five Likert scale that assesses the frequency with which each of these items occurs. A score of ‘1’ corresponds to ‘none of the time and a score of ‘5’ corresponds to ‘all of the time’. Total scores of the WEMWBS can vary from ‘14’ to ‘70’, higher the score the more the mental well-being.

Stressful conditions prevalent in respondents’ workplaces (hospitals or clinics) were defined as occupational stressors. Participants were asked to rate several work related stressors such as insufficient opportunities, uncertain future, Patient overload, long working hours, and illegitimate political or administrative pressure on a dichotomous scale (Yes/No).

Usage of five major food groups was noted over previous seven days on a recall basis. This were converted into servings by using a standardized conversion scale (Table S1). Finally, these figures were compared to USDA dietary guidelines 2010. (Table S1) Exercise was categorized as: ‘moderate’, ‘vigorous’ and ‘muscle strengthening’. American Heart Association (AHA) guidelines were used to estimate the exercise pattern of the respondents. According to AHA guidelines, the average requirement of physical exercise is 150 minutes per week of moderate or 75 minutes per week of vigorous exercise, or a combination of both [5]. For participants, exercise was noted in minutes per week, on a seven day recall basis. To simplify analysis and interpretation, vigorous exercise time was multiplied by two. Therefore making a cut off for combination of two activities was easier. Recommended exercise levels were then defined as 150 min/week. Detailed methods can be read in our previous publications [4,6]

**Ethics disclaimer:**
Ethical approval was sought and received from the Ethics Review Committee at CMH Medical College Lahore Cantt. All participants were assured of anonymity and written consent to participate was obtained. No individual finding were reported upon a particular subject.

References:

1. Vanvoorhis CRW, Morgan BL. Understanding Power and Rules of Thumb for Determining Sample Sizes. Tutorials Quant. Methods. 2007;3:43–50.

2. Clarke A, Friede T, Putz R, Ashdown J, Martin S, Blake A, et al. Warwick-Edinburgh Mental Well-being Scale (WEMWBS): validated for teenage school students in England and Scotland. A mixed methods assessment. BMC Public Health [Internet]. BioMed Central Ltd; 2011;11:487. Available from: http://www.biomedcentral.com/1471-2458/11/487

3. Taggart F, Friede T, Weich S, Clarke A, Johnson M, Stewart-Brown S. Cross cultural evaluation of the Warwick-Edinburgh mental well-being scale (WEMWBS) -a mixed methods study. Health Qual. Life Outcomes [Internet]. Health and Quality of Life Outcomes; 2013;11:27. Available from: http://www.hqlo.com/content/11/1/27

4. Waqas A, Ahmad W, Haddad M, Taggart FM, Muhammad Z, Bukhari MH, et al. Measuring the well-being of health care professionals in the Punjab: a psychometric evaluation of the Warwick–Edinburgh Mental Well-being Scale in a Pakistani population. PeerJ. PeerJ Inc.; 2015;3:e1264.

5. Haskell WL, Lee IM, Pate RR, Powell KE, Blair SN, Franklin BA, et al. Physical activity and public health: Updated recommendation for adults from the American College of Sports Medicine and the American Heart Association. Med. Sci. Sports Exerc. 2007. p. 1423–34.

6. Ahmad W, Taggart F, Shafique MS, Muzafar Y, Abidi S, Ghani N, et al. Diet, exercise and mental-wellbeing of healthcare professionals (doctors, dentists and nurses) in Pakistan. PeerJ [Internet]. 2015;3:e1250. Available from: https://peerj.com/articles/1250

Table 1: Food Exchange List

| **FOOD GROUP** | **COMPONENT** | **SERVING SIZE or EXCHANGE (E)** |
| --- | --- | --- |
| **Starchy**  **Food** | Roti**^2^** | 1 Roti (8 inches)=2E |
|  | Paratha***^,2^** | 1 Paratha (8 inches)=2E |
|  | Rice**^1^** | 1 medium plate containing 1 cup cooked rice=3E |
|  | Breakfast Cereals**^1^** | 1 medium bowl=1.33E |
|  | Bread**^1^** | 1 Slice=1E |
| **Meat, Fish & other Protein Source** | Red Meat**^1^** | 1 medium sized pieces(Boti)=3E |
|  | Chicken**^1^** | 1 medium sized pieces(Boti)=3E |
|  | Fish**^1^** | 1 med size fish=3E |
|  | Eggs**^1^** | 1 medium sized=1E |
|  | Pulses(Dal, Pea)**^1^** | 1 medium plate containing 1 cup of pulses= 2E |
| **Dairy** | Milk**^1^** | 236ml=1E |
|  | Yogurt**^1^** | 177.4g=1E |
| **Fruits & Vegetables** | Large sized fruits (e.g. mango or pineapple)**^1^** | 2 slices=1E |
|  | Medium sized fruits (e.g. apple or banana)**^1^** | 1 medium sized fruit=1E |
|  | Small fruits (e.g. plums)**^1^** | 2 small fruits=1E |
|  | Grapes & Berries**^1^** | 2 handfuls=1E |
|  | Fruit juices**^1^** | 118 ml=1E |
|  | Vegetables**^1^** | 1 medium plate containing 1 cup cooked vegetables=3E |
|  | One medium=2E | Potato (Baked)**^1^** |
|  | Salad leaves**^1^** | 1 dessert bowl= 1E |

***= Contains fat**

**Reference:**

# Krause's Food & Nutrition Therapy (12^th^ Edition)

1. FSANZ Online database: NUTTAB as well as “The New Traffic Light Guide” by the Diabetes Education and Assessment Program, Royal North Shore Hospital, Australia.
2. Waqas A, Ahmad W, Haddad M, Taggart FM, Muhammad Z, Bukhari MH, et al. Measuring the well-being of health care professionals in the Punjab: a psychometric evaluation of the Warwick–Edinburgh Mental Well-being Scale in a Pakistani population. PeerJ. PeerJ Inc.; 2015;3:e1264.
