# Supplementary material for: Exploring diet, exercise, chronic illnesses, occupational stressors and mental well-being of healthcare professionals in Punjab, Pakistan
Source: BMC Res Notes. 2017 Dec 19;10:745. doi: 10.1186/s13104-017-3096-5 (PMC5735512; doi:10.1186/s13104-017-3096-5)
Supplement: Supplementary file 2 — Additional file 2. Questionnaire used for interviewing participants. This document contains the questionnaire employed in present study. [file 13104_2017_3096_MOESM2_ESM.doc]

**“Diet, Exercise and Mental-Wellbeing of Doctors and other Healthcare Professionals of Pakistan”**

Age: _________ Sr. No: ________________

Gender: M / F Marital Status: _______________

Healthcare Profession: a) Doctor. *Specialty (Doctors & Dentists only):*_____________________

b) Nurse. Designation: ______________________

c) Pharmacist.

d) Physiotherapist.

e) Dentist.

**Q.1: How many times do you eat breakfast in a week?**

I eat breakfast_________ times/week

**Q.2: How many times do you eat fast food (Pizza, Zinger, Chips etc) in a week?**

I eat fast food _________ times/week

**Q.3: Most of the meals you eat are:**

a) Home-made.

b) Restaurant-made.

**Q.4: How many glasses of water you drink on average in a day? (1 glass of water= 250ml)**

I drink __________ glasses of water/day.

**Q.5: How many cups of coffee or tea you take in a day?**

Tea_________, Coffee_________.

**Q.6: Do you take any supplements (Iron or Vit.D etc)?**

a) No.

b) Yes, I take supplement for______________________

(P.T.O)

**[Fill in the number of respective food items you eat in a week *e.g. if you eat 3 slices of bread a day then you would write 21(3x7=21) in the column next to it to make a week’s total.*]**

| **FOOD GROUP** | **COMPONENT** | **Portion** | **YOU EAT IN A WEEK** |
| --- | --- | --- | --- |
| **Starchy**  **Food** | Roti (Phulka) | 1 Roti |  |
|  | Paratha | 1 Paratha |  |
|  | Rice | 1 medium plate containing 1 cup cooked rice |  |
|  | Breakfast Cereals | 1 medium bowl |  |
|  | White Bread | 1 Slice |  |
| **Meat, Fish & other Protein Source** | Red Meat | 1 med-piece(Boti) |  |
|  | Chicken | 1 med-piece (Boti) |  |
|  | Fish (White) | 1 med-piece |  |
|  | Fish (Oily) | 1 med-piece |  |
|  | Eggs | 1 medium sized |  |
|  | Pulses(Dal, Pea) | 1 medium plate containing 1 cup of pulses |  |
| **Dairy** | Milk | 250ml= 1glass |  |
|  | Yogurt | 250g= 1quarter (Pao) |  |
| **Fruits & Vegetables** | Large sized fruits (e.g. mango or pineapple) | 1 slice |  |
|  | Medium sized fruits (e.g. apple or banana) | 1 medium sized fruit |  |
|  | Small fruits (e.g. peach) | 1 small fruit |  |
|  | Grapes & Berries | 1 handful |  |
|  | Fruit juices | 250ml= 1glass |  |
|  | Vegetables | 1 medium plate containing 1 cup cooked vegetables |  |
|  | Potato (Baked) | One medium |  |
|  | Salad leaves | 1 dessert bowl |  |
| **Fat** | **Saturated:** Butter, Cream, Chocolate, Cakes, Chips etc. | | 1. Infrequently.   b) Often.  c) Very frequently. |
|  | **Un-saturated:** Almonds, Peanut butter, Olive oil, nuts etc. | | 1. Infrequently.   b) Often.  c) Very frequently. |
| **Sugar** | Cola drinks, sweets, biscuits etc. | | 1. Infrequently 2. Often 3. Very frequently |

Sr. No: *__________*

**“Diet, Exercise and Mental-Wellbeing of Doctors and other Healthcare Professionals of Pakistan”**

(All the information given by you would be kept with anonymity. In case of disclosure we shall take full responsibility. Encircle your choice.)

**Q.1: Have you been diagnosed with any of the illnesses listed below?** (*You can choose more than one options*)

a) None e) Visual defects like Myopia or Hyperopia.

b) Coronary Heart Disease. f) Back problems.

c) Hypertension. g) Other: - ____________________

d) Diabetes Mellitus.

[*Have you taken any life style changes to cope with the disease? a) Yes b) No*]

**Q.2: Do you do exercise as a part of your daily routine?**

a) No.

b) If Yes, then fill in the following details:

| **Type of Exercise** | **Duration**  **(mins)/Day** | **Days/Week** |
| --- | --- | --- |
| **Moderate-intensity aerobic** e.g. *walking fast, water aerobics, riding a bike on level ground or with few hills, doubles tennis, pushing a lawn mower, hiking, skateboarding, rollerblading, volleyball, basketball etc.* |  |  |
| **Vigorous-intensity aerobic** e.g. jogging or running, swimming fast, riding a bike fast, singles tennis, football, skipping rope, hockey, aerobics, martial arts. |  |  |
| **Muscle-strengthening activity** e.g. **lifting weights, working with resistance bands, doing exercises that use your body weight for resistance, such as push-ups and sit-ups, yoga etc. (1 set= 8-12 repeats)** | **Sets/day** |  |
|  |

**Q.3: Do you smoke?**

a) No.

b) Yes. (Specify the number: ________ /Day)

**Q.4: Do you go for your routine check-up to the dentist?**

a) No.

b) Yes. (Number of Visits: _________ /Year)

**Q.5: How many hours on average you sleep in a day?**

_________ Hours/Day.

**Q.6: How many hours do you work in a week?**

_________ Hours/Week.

**Q.7: How many hours do you think you should work in a week in order to perform your duties comfortably?**

_________ Hours/Week.

**Q.8: What is your:-**

Weight (Kg) = ___________ Height= (Ft): __________ (In): _________ or __________ (meter).

**Q.9: In your routine do you get to spend time with your family as much as you desire for?**  a) Yes. b) No.

(P.T.O)

**Q.10: Do you have easy access to social, medical, educational etc. services?**

**a)** Yes.

**b)** No.

**Q.11: Your monthly income falls in which category (Per Month)?**

a) Low Income (≤ Rs. 8, 500)

b) Lower-Middle Income (≥ Rs 8, 501 and ≤ Rs. 33, 000)

c) Upper-Middle Income (≥ Rs. 33, 001 and ≤ Rs.102, 000)

d) High-Income (> Rs.102, 001)

**Q.12: Do you think you are treated in a way you deserve to be treated in the society?**

**a)** Yes.

**b)** No.

***(You could skip this question if you want to) Q.13: Do you use any of the substances mentioned. Alcohol, opioids, methamphetamine etc.***

**a)** No. **c)** I used to in the past but not now.

**b)** Yes.

**Q.14: Do you feel under stress related to your work? If YES then which form of stress you encounter the most: *(Use number scale to show your emphasis.” 1” being the lowest and “5” being the highest You could choose more than one options.)***

a) No, I do not feel under stress.

a) Long working hours. 1, 2, 3, 4, 5.

b) Patient overload. 1, 2, 3, 4, 5.

c) Uncertain future and limited opportunities to prosper. 1, 2, 3, 4, 5.

d) Insufficient rewards or acknowledgement of your work. 1, 2, 3, 4, 5.

e) Illegitimate *political*, *administrative* etc. pressure. 1, 2, 3, 4, 5.

f) None of the above. (Other: ________________________)

**Q.15: If you could go back in time, would you choose a different profession?**

**a)** Yes.

**b)** No.

*Please encircle the box that best describes your experience of each over the last 2 weeks*

| **STATEMENTS** | **None of the time** | **Rarely** | **Some of the time** | **Often** | **All of the time** |
| --- | --- | --- | --- | --- | --- |
| **I’ve been feeling optimistic about the future** | **1** | **2** | **3** | **4** | **5** |
| **I’ve been feeling useful** | **1** | **2** | **3** | **4** | **5** |
| **I’ve been feeling relaxed** | **1** | **2** | **3** | **4** | **5** |
| **I’ve been feeling interested in other people** | **1** | **2** | **3** | **4** | **5** |
| **I’ve had energy to spare** | **1** | **2** | **3** | **4** | **5** |
| **I’ve been dealing with problems well** | **1** | **2** | **3** | **4** | **5** |
| **I’ve been thinking clearly** | **1** | **2** | **3** | **4** | **5** |
| **I’ve been feeling good about myself** | **1** | **2** | **3** | **4** | **5** |
| **I’ve been feeling close to other people** | **1** | **2** | **3** | **4** | **5** |
| **I’ve been feeling confident** | **1** | **2** | **3** | **4** | **5** |
| **I’ve been able to make up my own mind about things** | **1** | **2** | **3** | **4** | **5** |
| **I’ve been feeling loved** | **1** | **2** | **3** | **4** | **5** |
| **I’ve been interested in new things** | **1** | **2** | **3** | **4** | **5** |
| **I’ve been feeling cheerful** | **1** | **2** | **3** | **4** | **5** |

Warwick-Edinburgh Mental Well-Being Scale (WEMWBS)

© NHS Health Scotland, University of Warwick and University of Edinburgh, 2006, all rights reserved.
